# Wet anaerobic digestion of organic fraction of municipal solid waste: experience with long-term pilot plant operation and industrial scale-up

**DOI:** 10.1007/s00449-023-02958-2

**Published:** 2024-01-03

**Authors:** Omar Anaya-Reza, María F. Altamirano-Corona, Germán Basurto-García, Héctor Patricio-Fabián, Sergio A. García-González, Elias Martinez-Hernandez, Alfonso Durán-Moreno

**Affiliations:** 1 Chemical Engineering Department, Faculty of Chemistry, Universidad Nacional Autónoma de México, Circuito de La Investigación Científica, 04510 Mexico City, Mexico; 2 Biomass Conversion Division, Instituto Mexicano del Petróleo, 07730 Mexico City, Mexico

**Keywords:** Wet anaerobic digestion, Biogas, OFMSW, Pilot plant, Scaling-up

## Abstract

This paper presents the analysis of a pilot anaerobic digestion plant that operates with organic fraction of municipal solid waste (OFMSW) from a wholesale market and can treat up to 500 kg d^−1^. The process was monitored for a period of 524 days during which the residue was characterized and the biogas production and methane content were recorded. The organic load rate (OLR) of volatile solids (VS) was 0.89 kg m^−3^ d^−1^ and the Hydraulic Retention Time (HRT) was 25 d during the process. The yield was 82 Nm^3^ tons OFMSW^−1^ biogas, equivalent to 586 Nm^3^ tons CH_4_ VS^−1^. The results obtained in the pilot plant were used to carry out a technical–economic evaluation of a plant that treats 50 tons of OFMSW from wholesale markets. A production of 3769 Nm^3^ d^−1^ of biogas and 2080 Nm^3^ d^−1^ of methane is estimated, generating 35.1 MWh d^−1^ when converted to electricity.

**Supplementary Information:**

The online version contains supplementary material available at 10.1007/s00449-023-02958-2.

## Introduction

Fossil resources account for more than 80% of global energy consumption. According to the International Energy Agency, this sector is expected to increase carbon dioxide (CO_2_) emissions from 50 in 2030 to 80% in 2050. As a result, the environmental problems associated with the use of fossil fuels have been of concern to the international community. As a result of the energy and climate crisis, renewable energies are expected to become a viable alternative [1]. In Mexico, the generation of clean energy from renewable resources represents <16%. The total installed generation capacity in the country is 53,114 MW, ranking Mexico as seventh in the world. However, it is estimated that more than 20,000 MW of additional capacity would be needed in the future to cope with the expected growth in demand [2]. Wastes and residues abundant in the country can be an alternative energy source to meet such increasing demand. An often overlooked source of residues are the wholesale markets. For example, Mexico City hosts the second largest wholesale market in the world [3], where 13,073 tons of municipal solid waste (MSW) are generated daily, of which about 18% is not collected and is disposed of in illegal dumps, burning in the open air or handled by an informal waste sector [4]. The municipal solid waste management system of the city consists of 12 transfer stations, 3 separation and 8 composting plants, and 5 landfills located outside of the city [5].

Waste-to-energy (WtE) technologies, which can produce heat and electricity by converting waste through a thermochemical or biochemical process, have received attention to increase resource efficiency, alleviate environmental pressures and generate greater economic benefits. Common examples include gasification, incineration, refuse-derived fuel production and anaerobic digestion [6]. The challenge of managing this solid waste while ensuring environmental protection has led to the need to develop appropriate new and innovative treatment options that allow OFMSW, based on the concept of a circular economy, to be used for other purposes and help alleviate the waste problem. One such treatment option is the use of anaerobic digestion (AD) [7].

In developing countries, anaerobic digestion technology plays an essential role, not only in waste management, but also in responding to energy demand, especially in remote rural areas not connected to the electrical grid. This allows these nations an opportunity to economically supply energy and improve quality of life [8]. The Deutsche Gesellschaft für Internationale Zusammenarbeit (GIZ) has supported the development and dissemination of technology in the region. Most of the digesters were developed under a 100% subsidy model but were not accompanied by specific training and monitoring. For this reason, the study has not been followed up, so projects were abandoned by users [9]. Although this technology has been in use for an extensive period in developing countries, a more exhaustive analysis is still required to fully understand the context, challenges, and possibilities from a Latin American perspective [8].

In recent decades, the development of AD plants has been proposed as a strategy to achieve both environmental pollution mitigation and energy independence by utilizing waste streams such as livestock manure and OFMSW to produce biogas and biofertilizer [10]. Biogas and nutrient-rich digestate produced by AD plants generate significant environmental savings, specifically in terms of mitigating greenhouse gas (GHG) emissions [11]. This technology could reduce adverse impacts and improve the sustainability of food waste management to a greater extent than landfilling and incineration. A promising finding is that most impact categories, excluding freshwater consumption, would be reduced by more than 50% in 2050 compared to 2020 values [12]. The anaerobic digestion process can be carried out in a wet and dry way. In wet anaerobic digestion, the dry matter content in the substrate is relatively low, generally less than 15% dry matter. In this case, the organic matter is kept in a wet or liquid state before entering the reactor compared to dry anaerobic digestion [13]. In general, wet anaerobic digestion is more commonly used than dry anaerobic digestion, because it is easier to control and offers higher biogas production efficiency.

The main barriers that limit AD of OFMSW for energy production in developing countries such as Mexico have been the lack of an efficient separation technique, inadequate technical knowledge, or the currently perceived complexity of the process operation as well as insufficient funding, which have resulted in the accumulation of immense amounts of waste. Other limitations and constraints are the long start-up of reactors, relatively long stabilization times and the occurrence of toxic and inhibitory effects caused by some compounds, especially when OFMSW are not adequately separated from inorganic wastes [14]. Therefore, AD has been implemented in Mexico focusing mainly on small-scale biodigesters, commonly known as domestic biodigesters or household biodigesters [15].

There are some publications describing the experience of the anaerobic digestion process at pilot and industrial scale [16–18]. There are authors like Tsydenova [5], who have carried out studies on anaerobic digestion in Mexico. They conclude a possible economically viable option and a favorable environmental impact due to the reduction of methane released into the atmosphere. Campuzano et al. [19] has carried out different anaerobic digestion studies in Mexico City. However, there are few papers reporting treatment of OFMSW at relatively high scale and over long periods of time [20–22]. In this work, data for a Pilot Biogas Producing Plant (referred to as 3PBg) having the capacity to treat up to 500 kg of substrate per day is analyzed and discussed. In this plant, organic waste from a wholesale market in Tultitlan, located in the north of the metropolitan area of Mexico City, was used as substrate. The work includes the description of the system, characterization of the OFMSW and performance evaluation, assessing 3PBg for an urban environment. The pilot plant data were used to evaluate a scaled version of this plant with greater treatment capacity and power generation presented here to determine the profitability for the treatment of waste in wholesale markets.

## Materials and methods

### Characterization

The organic waste processed in the 3PBg was collected at the Central of Abastos Tultitlán, Mexico, following the sampling protocol established in the Mexican standard NMX-AA-015-1985. The substrate was physically characterized to classify and quantify the identifiable components in the sample, based on the NMX-AA-22-1985 standard. The physicochemical parameters included in the analysis of the substrate, the sludge inoculum and the digestate included moisture and total solids (TS) (APHA-2540-B); volatile solids (VS) and fixed solids (FS) (APHA-2540-E); chemical oxygen demand (COD) (APHA-5220-D); ammoniacal nitrogen (N-NH_4_) (APHA-4500-NH_3_-C and APHA-4500-NH_3_-E); total phosphorus (P total) (APHA-4500-P-B4 and APHA-4500-P-C) applying standard methods of the American Public Health Association [23]; volatile fatty acids (VFA) [24] and; alpha factor (*α*), intermediate alkalinity caused by VFA, and partial alkalinity caused by HCO_3_ species [25].

### Pilot plant operation

**Fig. 1:**
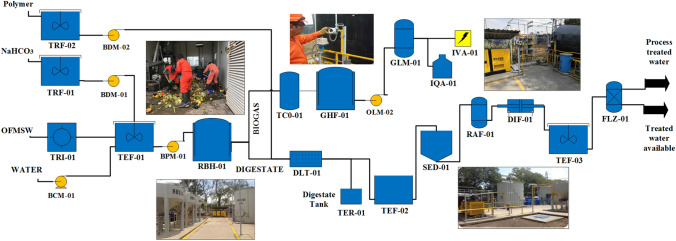
Flow diagram of 3PBg. The equipment list is as follows: TRF-01 Sodium Bicarbonate Dissolving Tank; TRF-02 Polymer dissolution tank; TRI-01 Shredder; BCM-01 Drinking water feed pump; BDM-01 Sodium bicarbonate dosing pump; BDM-02 Polymer dosing pump; TEF-01 Homogenization tank; BPM-01 Digester feed pump; RBH-01 Anaerobic biodigester; TCO-01 Condensate trap; GHF-01 Gas reservoir; OLM-02 Biogas blower; GLM-01 Gas reservoir; OLM-02 Biogas blower; GLM-01 Biogas purifier; IQA-01 Gas burner; IVA-01 Electric generator; DLT-01 Sludge dehydrator; TEF-02 Wastewater storage tank; TER-01 Digestate storage container; SED-01 Settling equipment; RAF-01 Filter type anaerobic reactor; DIF-01 Air diffusor; TEF-03 Treated water tank; FLZ-01 Zeolite filter

The pilot plant has a wet anaerobic digestion reactor with a capacity of 25 m^3^ with continuous agitation and is designed to operate 24 h per day, 360 days a year with a semi-automatic control. Manual operations include parameter monitoring, OFMSW collection, feeding to the wet anaerobic digester and digestate extraction, activities that are carried out every third day. The sludge inoculum was acclimatized for 4 months. In this period, the digester was operated at 37 °C, and it was fed mainly with vegetable residues. Figure 1 shows the flow diagram of the 3PBg process.

Pretreatment of the waste is an important step for proper feeding and operation of the biodigester. Mechanical pretreatment is applied to reduce the substrate particle size to 5 mm and maintain a concentration of 10% total solids with the addition of water (potable water or treated wastewater recirculated from the process). In the process of pilot plant operation, a wet anaerobic digester increases the contact area of the substrate with the inoculum, which allows a more efficient degradation and enhances biogas generation. Nevertheless, undesirable components such as large bones, plastics, and metals, which can cause blockages or damage to mechanical equipment, are removed from the organic feed material. After this separation step, shredded OFMSW is discharged by gravity to a homogenization tank, where 216 kg of sodium bicarbonate solution (6%) is added per ton of OFMSW. The pH was maintained between 6.0 and 7.0 mainly to achieve high methane production according to various studies [26–28]. Temperature can be a fundamental parameter to achieve greater biogas production. Since the microorganisms in the sludge inoculum are mesophilic in nature, the whole process was carried out under mesophilic conditions to maintain the economics of the process. The digester was fed with a maximum volume of granular anaerobic sludge inoculum of 21 m^3^, coming from a continuous stirred tank anaerobic reactor (CSTR) that treats effluents from the brewing industry located in Mexico City, having a hydraulic retention time (HRT) of 25 days for OFMSW. Therefore, anaerobic digestion is carried out in a single stage at a temperature of 35 °C and maintained the same with mechanical agitation. A sample of diluted OFMSW was taken to determine the pH. At pH values below 7, sodium bicarbonate 0.1 N (NaHCO_3_) is added until a neutral pH is reached to guarantee adequate feeding conditions for this type of system. The biogas produced passes through a condensate trap and treated by two H_2_S removal columns (to reach up to 3 ppm) by chemical reaction with Fe_2_O_3_. Fe_2_O_3_ is used, because an acid–base reaction is carried out, where Fe_2_O_3_ is the base and H_2_S is the acid. In this reaction, sulfur is obtained, which is oxidized forming elemental sulfur. In the ADP study, around 1000 ppm of H_2_S was produced. When the reactions mentioned above occur, a reduction to 3 ppm is achieved. That is, a 99.7% elimination is achieved. The biogas is then used as a fuel in an electric generator, which has an internal combustion engine. When this equipment is not in service or fails, the biogas is burned. The daily determination of the composition of the biogas was carried out by means of a specialized portable analyzer equipment (STATUS SCIENTIFIC CONTROLS, model: PGD3-IR), which had the capacity to detect methane (CH_4_), Carbon Dioxide (CO_2_), Sulfide Hydrogen (H_2_S) and Oxygen (O_2_). Therefore, the determination of H_2_ was not possible.

The digestate produced as a by-product in the wet anaerobic digester is dehydrated through a combination of dewatering and drying units. The process also has a wastewater treatment plant that consists of equipment that operates sequentially to treat wastewater resulting from the dewatering unit. The purpose of this is to obtain treated water for recycling as dilution water in the process or for cleaning services.

Feeding to 3PBg was variable, ranging from 0 to 575 kg OFMSW d^−1^ (average 387 ± 94 kg d^−1^) considering a time period of 324 days. The wastes fed to the reactor consisted mainly of vegetables, fruits, leachate, and yard waste. An Anderson–Darling statistical test was performed to verify the type of data distribution for COD, moisture, and ST.

## Results and discussion

### Operational experience in the 3PBg

In this section, our objective is to contribute to future operational processes, so it is essential to highlight the experience acquired in the management of the pilot plant*.* The 3PBg is a pilot plant that requires control equipment to maintain greater stability during the process. The plant operated with a sludge inoculum that was not adapted to the OFMSW, so the digestion took time to stabilize. First, the appropriate operating conditions (temperature, pH, agitation, HRT, solids concentration, microorganism adaptation, safety measures and gas monitoring) had to be found, considering the climatic variations of the site. Moreover, the composition of the waste supplied varied according to the season and sometimes, the feeding quantities were altered because the supplier of the waste did not comply with the scheduled dates. On the other hand, some members of the population do not separate organic waste from inorganic waste. When the waste arrives at the pilot plant, it must be separated manually so as not to damage the equipment and for the anaerobic digester to function properly. However, a good separation is not achieved. To solve this, it is recommended that a proper separation plant is installed and to locate the pilot plant in a strategic site to ensure continuous feeding.

### Substrate characterization and biogas yield

**Fig. 2:**
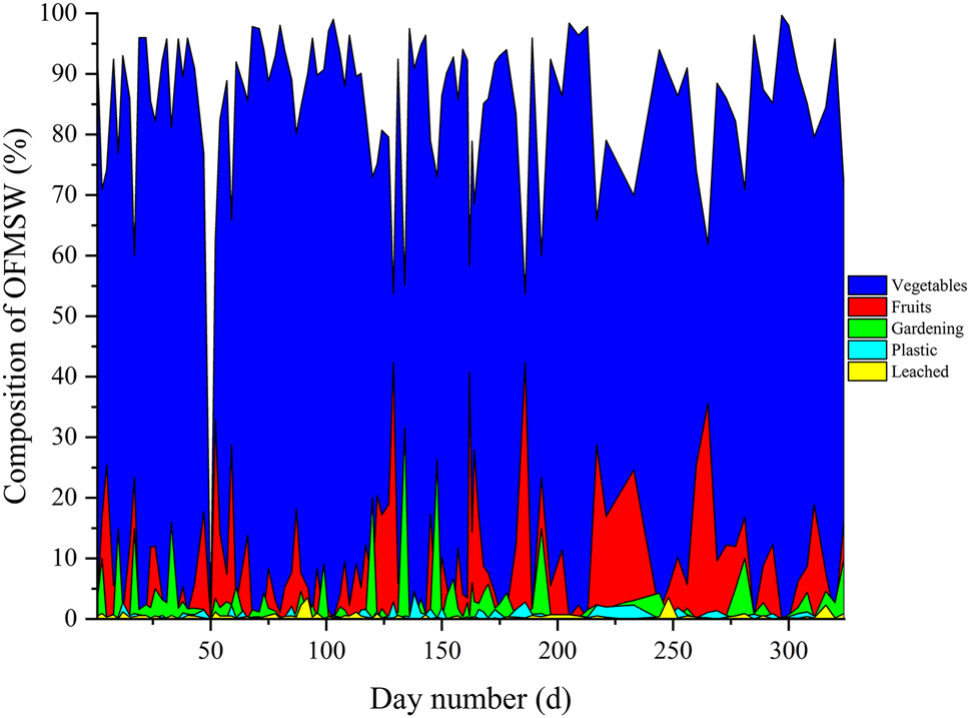
Characterization of the main OFMSW compounds of the wholesale market

Thirteen by-products were categorized, of which 6 were treatable by anaerobic digestion (vegetables, fruits, meats, gardening, leachate and other organic), 6 were inorganic and 1 by-product (sanitary waste). The characterization of the OFMSW received at the pilot plant from the wholesale market is presented in Fig. 2. Composition shows variations depending on the day.

The characteristics for OFMSW and sludge were determined to know the behavior within the process. Table 1 summarizes the average values over two periods of time. The period in which the sludge takes time to adapt to the substrate (from day 1 to 120) is Period I. Period II comprises from day 121 until the end of the process at 3PBg (Period II).

**Table 1:** Characteristics of OFMSW and sludge inoculum in Period I and Period II

Characteristic	Period I	Period II	
	OFMSW	OFMSW	Sludge
Temperature (°C)	30 ± 4.8	31 ± 6.1	–
COD (g O_2_ L^−1^)	50 ± 1.3	130 ± 35	56 ± 36
Moisture (%)	86 ± 0.14	92 ± 61	98 ± 1.2
TS (%)	13 ± 0.56	7.5 ± 1.7	1.8 ± 1.2
VS (%)	12 ± 1.7	6.2 ± 1.6	1.0 ± 1.1
COD VS^−1^	–	0.79 ± 0.58	–
TKN (g L^−1^)	2.0 ± 0.14	2.1 ± 0.62	1.5 ± 1.4
N-NH_4_ (g N L^−1^)	0.20 ± 0.01	0.38 ± 0.24	0.95 ± 0.18
P total (g P L^−1^)	–	13 ± 20	12 ± 16
Alkalinity (g CaCO_3_ L^−1^)	4.5 ± 0.2	3.6 ± 1.2	7.6 ± 1.6
VFA (g CH_3_COOH L^−1^)	7.6 ± 0.7	5.4 ± 2.3	0.39 ± 0.35

*TKN* total Kjeldahl nitrogen

During the process, the operating conditions were maintained according to the values proposed by Panigrahi & Dubey [29], such as alkalinity, pH and temperature. Besides, the following recommendations by Holliger et al. [30] were followed for the inoculum: N-NH_4_ + <2.5 g L^−1^; 7.0 < pH < 8.5; alkalinity >3 gCaCO_3_; VFA < 1.0 gCH_3_COOH L^−1^. The amount of total P for OFMSW and sludge inoculum, in addition to VS for sludge, presented dispersion and a greater variability of the data. Total Kjeldahl Nitrogen refers to the measure of the total organic matter content in OFMSW and sludge. The amount of TKN in OFMSW depends on the composition of the residues and can vary widely, however, there are studies that obtained similar values [31, 32]. The COD/VS ratio means that for every gram of volatile organic matter suspended in a substrate, there is expected to be approximately 0.8 g of COD. The ratio is low, so it could indicate the presence of organic matter that is difficult to biodegrade or inhibitors in the substrate.

It is important to maintain a proper balance of nutrients in anaerobic digestion to ensure optimal yield and avoid problems such as inhibition of the process. Therefore, the amounts of total phosphorus need to be adjusted. In addition, the optimal range of the TS percentage may vary depending on the type of substrate and the specific conditions of the anaerobic digestion process. In general, a TS percentage between 6 and 10% is considered adequate for efficient anaerobic digestion. Aboudi et al. [33] found that the highest percentage of volatile solids is 8% using sugar beet by-products and cattle manure. Yi et al. [34] obtained high biomethane production yields considering 5–20% of TS, using OFMSW as a substrate. They noted that increasing TS within this range also increased the yield. Although the amount of TS in 3PBg is adequate, even higher yields of biomethane per substrate could be achieved. Another important factor is the VS, because they are the organic fraction of the substrate that is converted into gas (mainly methane) during the anaerobic digestion process. The VS concentration used in this investigation was around 6.21%. The VS was on average 96% for Period I and 83% for Period II, of the TS content. This indicates a potential for organic transformation of these substrates during the AD processes. In general, adequate volatile fatty acid levels in anaerobic digestion are in the range of 1–4 g L^−1^. On average, VFA values were found to be slightly higher than recommended, especially in the start-up stage (Period I). However, during the process, the pH was maintained between 6.0 and 7.0. In addition, an insufficient level of alkalinity can cause a decrease in pH and an inhibition of methanogenic microorganisms, which negatively affects biogas production. Desirable alkalinity values are between 2 and 6 g L^−1^. This range provides a good balance in the system and helps to avoid drastic changes in pH. Thus, the alkalinity values for OFMSW remained stable, however, the alkalinity in the sludge inoculum was high. Furthermore, VFA Alkalinity^−1^ ratio <0.9 is desirable for the microbial community to be balanced and avoid acid inhibition. This caused difficulty during the process of maintaining the desired pH values [35].

Table 2 presents the chemical characteristics of organic municipal solid waste (OFMSW) in developing countries. Although some of these countries are geographically distant from Mexico, it is interesting to note that some of the values in Table 2 are comparable to the results obtained in our research, which are detailed in Table 1.

**Table 2:** Chemical characteristics of OFMSW in developing countries

Country	City	pH	Humidity (%)	TS (%)	VS (%)	References
Colombia	Bucaramanga	–	84 ± N/A	16 ± N/A	15 ± N/A	[19]
India	Kerala	6.2	81 ± N/A	19 ± N/A	17 ± N/A	[19]
India	Kashmir	–	50 ± 7	–	19 ± 3	[36]
India	–	5.8	82 ± 1	18 ± 2	17 ± 0.02	[37]
Bangladesh	Chittagong	8.2	62 ± N/A	–	54 ± N/A	[38]
Bangladesh	Dhaka	8.6	70 ± N/A	–	71 ± N/A	[38]
Sri Lanka	Kaduwela	–	–	26 ± 1	85 ± 1	[39]
Malaysia	–	–	15 ± N/A	–	69 ± N/A	[40]
Mexico	Mexico city	–	70 ± N/A	29 ± N/A	22 ± N/A	[19]

*N/A* no available

Using the same substrate and sludge, a Biochemical Potential of Methane (BMP) test was performed. The results present a similar yield (78 NLkg^−1^ OFMSW) to the average obtained in the pilot plant (NLkg^−1^ OFMSW), as well as TS, VS, VFA, and Alkalinity. On the other hand, a greater amount of organic matter present in BMP was obtained. This may indicate a higher level of organic load in the samples. The results are presented in the Supplementary Material.

**Fig. 3:**
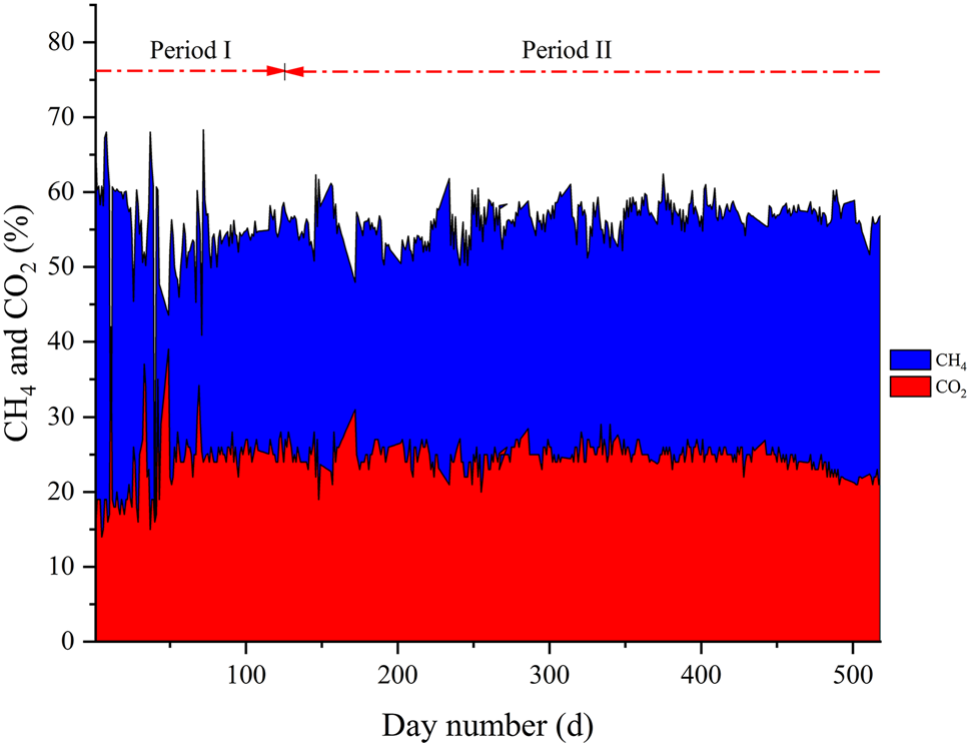
Percentage of biomethane and carbon dioxide in the biogas

In 3PBg, the biogas composition was more stable after 75 days. This is because there were feeding interruptions, and the inoculum was not yet adapted to the substrate. The value of average produced methane shown in Fig. 3 is lower compared to other studies [41–43]. However, similar values have been obtained using residues with a high proportion in fruits and vegetables [21, 44]. In this way, it can be observed that after 100 days, the production of CH_4_ and CO_2_ begins to stabilize.

**Fig. 4:**
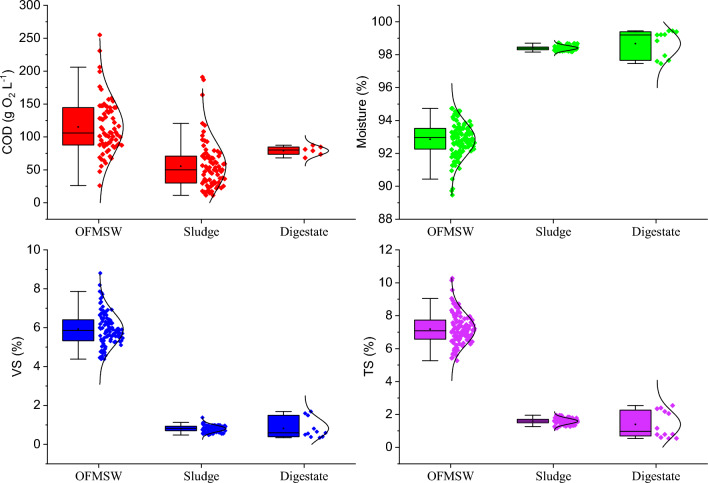
Average characterization of influents and effluents. The *bars* represent the standard deviation

From the Anderson–Darling test (in Fig. 4 for period II), a normal distribution was found for COD, VS, TS and moisture, with a 90% confidence interval, determined for OFMSW, sludge inoculum and digestate. This means that the values are distributed symmetrically around a central value, in this case, the average.

During period II, a greater amount of biogas is produced and the maximum capacity of each of the average yields can be appreciated for the most part. To obtain COD and VS removal, the methodology of Pavi et al. was carried out [45]. The specific digester productivity (G_CH4_) was determined according to Montecchio et al. [46]. This value is mentioned in Table 3 and refers to the amount of biogas produced per unit volume of the digester and per unit time. This measurement is important to evaluate the efficiency and performance of an anaerobic digestion system. Similarly, the HRT value is mentioned, which refers to the average time that the organic substrate remains inside the digester [47].

**Table 3:** Performance statistics for the pilot plant from day 121 to day 524

Measurements	Value	Units
Operational period	403	days
COD_removal_	51 ± 21	%
VS_removal_	64 ± 36	%
VS TS^−1^	82 ± 0.03	%
Methane	57 ± 2.3	%
Methane yield	580 ± 230	Nm^3^ CH_4_ ton VS^−1^
Methane yield	180 ± 32	Nm^3^ CH_4_ ton VS^−1^_removed_
Methane yield	18 ± 0.31	Nm^3^ CH_4_ ton OFMSW^−1^
Biogas yield	83 ± 18	Nm^3^ Biogas ton OFMSW^−11^
Volumetric daily biogas production	0.32 ± 0.25	Nm^3^_biogas_ m^−3^_digester_ d^−1^
OLR	0.89 ± 0.30	kg VS m^−3^ d^−1^
G_CH4_	1178	L m^−3^ d^−1^
HRT	25	d
Electricity yield	0.03 ± 0.02	kWh kg OFMSW^−1^
Total mass of OFMSW added	34,248	kg
Temperature	28 ± 3.8	°C
Alpha (*α*)	0.87 ± 0.08	

Dhar et al. [48] mentions a lower VS reduction of 55.7 and 60.7% when evaluating the yield of 2 anaerobic digesters of municipal solid waste. Petracchini et al. [41] report COD and VS removal of 75 and 95%, respectively, for a first-stage process, using an organic loading rate (OLR) and similar substrates. Therefore, high yield (%) was found especially in COD removal efficiency considering the quality of the digestate after the anaerobic process and biogas production. Besides, the methane yield (Nm^3^ CH_4_ ton VS^−1^) is higher than some other studies using two-stage anaerobic process [45, 49].

There are commercial options that can be classified into dry and wet systems based on TS, and the number of stages. In this case, 3PBg presents similar yields than these options, as Valorga (80–180 m^3^ of biogas ton OFMSW^−1^) or Waasa (100–150 m^3^ of biogas ton OFMSW^−1^) [50]. However, the resulting energy yield is low (kWh kg OFMSW^−1^) compared to values reported by Mu [51] and Ragazzi [52] that are between 0.15 and 0.30 kWh per ton of waste treated. For this study, the feeding of OFMSW to 3PBg varies according to the waste that was collected. Thus, it is difficult to determine an estimate of a plant, which treats a larger amount of waste. The capacity of 3PBg is 500 kg d^−1^. If it is considered that this amount is fed daily throughout the year, the total annual electrical energy consumption of the plant is 65 MWh. Nevertheless, considering a constant yield (82.7 Nm^3^ of Biogas ton OFMSW^−1^), the electricity generation would be 23 MWh year^−1^, i.e., the electricity consumption is higher than the pilot plant would generate. To achieve a balance between energy generated and consumed, the yield must be 237 Nm^3^ of Biogas ton OFMSW^−1^.

**Fig. 5:**
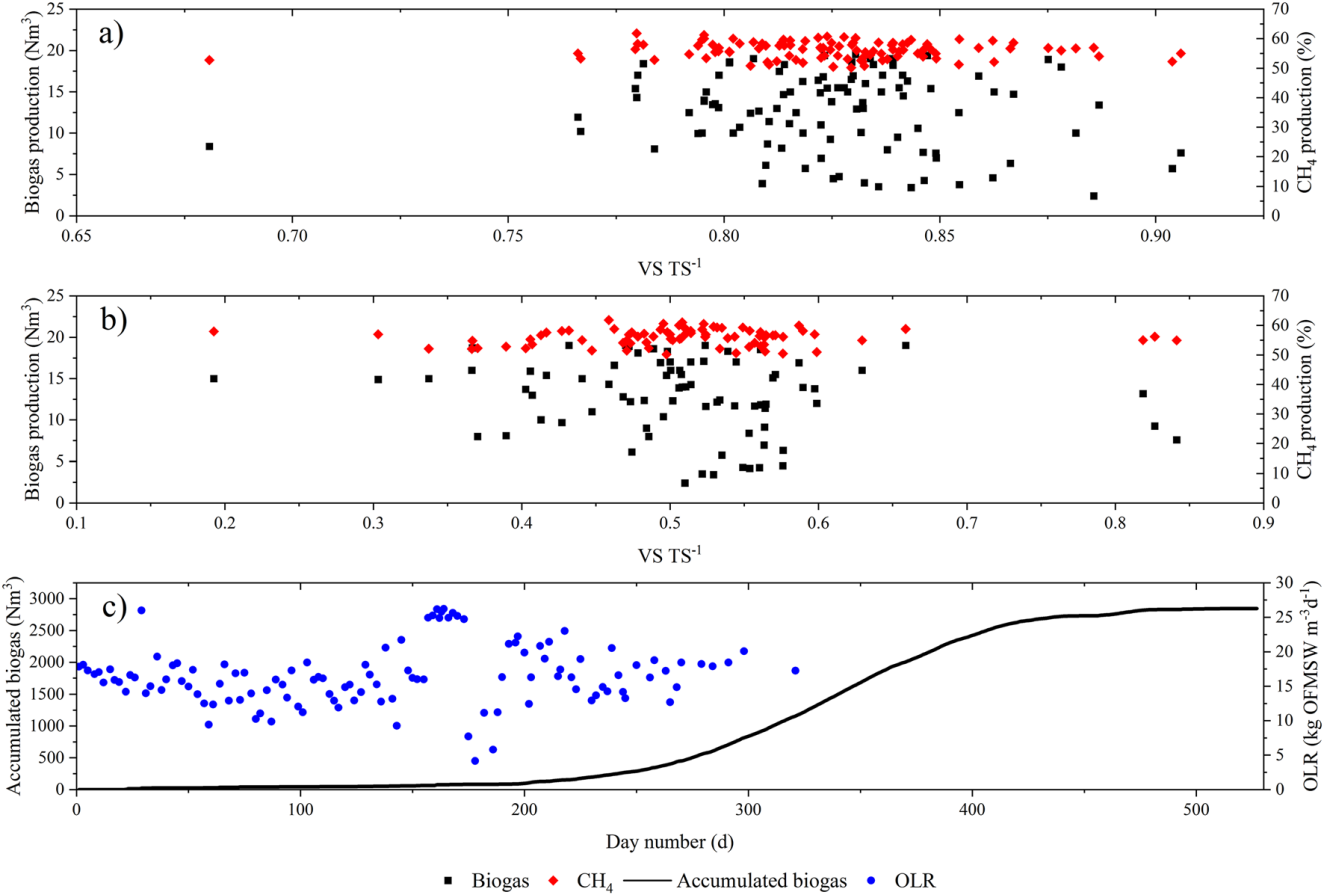
Biogas and biomethane production versus VS TS^−1^ for (**a**) OFMSW and (**b**) sludge. (**c**) OLR to digester and biogas accumulated

Another important value is the alkalinity index alpha (*α*), which shows the ratio of bicarbonate alkalinity to total alkalinity. A magnitude of alpha close to unity indicates that the wet biodigester is operating stably. Determination and control of alkalinities are necessary for efficient AD. Alpha (*α*) index corresponds to the ratio of bicarbonate, or partial alkalinity, to total alkalinity [53]. Besides, The VS TS^−1^ ratio indicates the amount of organic content in the substrate. The substrates with a higher VS TS^−1^ ratio contain more organics and are more suitable for biogas production [54]. For this study, the sludge inoculum that was fed to the digester only at the start of the operation is considered. However, sludge inoculum samples at different times were extracted from the digester for analysis of volatile and total solids. In addition, with the extracted sample, the reactor was purged, and the pH and alkalinity were measured inside the digester. All this is to review the operating parameters and the stability of the process. Similarly, substrate with a VS TS^−1^ ratio greater than 50% is considered to have a high organic content, suitable for anaerobic digestion for biomethane production [55]. In Fig. 5a, this ratio is shown for waste, while in Fig. 5b, the ratio is shown for sludge inoculum. On average, the VS TS^−1^ ratio for OFMSW is 83% (SD ± 0.03) and for the sludge is 51% (SD ± 0.11). Therefore, for this study, it can be considered that an adequate substrate was used (value shown in Table 3), and a sludge inoculum that during the process remains within acceptable limits for biogas production. These values indicate that more methane can be produced, and more energy generated while achieving greater stability in the process.

Residue feeding characteristics varied depending on the season of the year. The value of VS as low as 4% was determined during spring and summer. Whereas, in autumn and winter the amount increased considerably along with the amount of carbohydrate-rich residues. In this work, an average OLR of 0.89 kg VS m^−3^ d^−1^ (SD ± 0.30) and 15 kg OFMSW m^−3^ d^−1^ (SD ± 3.9) shown in Fig. 5c were obtained. Petracchini [41] report a very similar VS-related average OLR value of 0.8–1.28 kg VS m^−3^ d^−1^. However, they also mention some other values around 5 kg VS m^−3^ d^−1^. Rodríguez-Pimentel et al. [49] and Wang et al. [54] report very similar results at 2.5 kg VS m^−3^ d^−1^, while Pera et al. [47] values higher than 10 kg VS m^−3^ d^−1^. Besides, OLR with respect to kg OFMSW m^−3^ d^−1^ is higher for this study than values shown by Grimber et al. [21]. However, the yields are similar and to the results obtained by Walker et al. [22], with values of 446 and 596 m^3^ ton VS^−1^, respectively.

### Preliminary evaluation of the development of a scale anaerobic digestion plant

#### Main technical specifications

The data obtained from the pilot plant were used for a preliminary evaluation of a larger scale anaerobic digestion plant (ADP) for the treatment of OFMSW from wholesale markets in Mexico City. In ADP, the same equipment as 3PBg is considered, including the integration of heat that mainly comes from a turbine that takes advantage of the use of steam from the sludge dryer (DLT-01). A Sludge Dryer is used to represent an equipment resource typically used to host a drying procedure with a main objective to dry sludge material. Their purpose is to reduce sludge volume by decreasing the liquid content and increasing the solid content of sludge. The technoeconomic evaluation of ADP assumed a capacity to treat 50 tons of OFMSW per day, obtaining income from the electrical energy generated, tipping fee and the sale of digestate as a fertilizer. In this evaluation, only the anaerobic digestion process was considered and not the wastewater treatment, because there are already treatment plants in the wholesale market. The process evaluation was carried out using the process simulation software SuperPro Designer®.

The principle of similarity was used to scale up the digestion plant process and this principle refers to the relationship that exists between physical systems and their size, which are basic in the scale up of physical and chemical processes in the plant. Table 4 shows a summary of the technical specifications of the main equipment used.

**Table 4:** Main technical specifications of the pilot plant equipment

Equipment	Technical specifications (500 kg)	Technical specifications (50 tons)^a^
TRF-01	*V* = 0.088 m^3^; *H* = 0.7 m; *D* = 0.4 m	*V* = 1 m^3^; *H* = 2.25 m; *D* = 0.752 m
TRF-02	*V* = 0.088 m^3^; *H* = 0.7 m; *D* = 0.4 m	*V* = 1 m^3^; *H* = 2.25 m; *D* = 0.752 m
TRI-01	Ca = 250 kg h^−1^; *P* = 11.0 kW	Ca = 2083 kg h^−1^; *P* = 102.9 kW
BDM-01	Ca = 0.002 m^3^ h^−1^; *P* = 0.06 kW	Ca = 0.02 m^3^ h^−1^; *P* = 0.0004 kW
BDM-02	Ca = 0.004 m^3^ h^−1^; *P* = 0.06 kW	Ca = 0.01 m^3^ h^−1^; *P* = 0.0001 kW
BPM-01	Ca = 0.5 m^3^ h^−1^; *P* = 0.36 kW	Ca = 2.11 m^3^ h^−1^; *P* = 0.0004 kW
RBH-01	*V* = 25.5 m^3^	*V* = 1200 m^3^
DLT-01	Ca = 1.67 kg h^−1^	Ca = 1844 kg h^−1^
GHF-01	Ca = 6 m^3^	Ca = 50 m^3^
GLM-01	*D* = 0.305 m; *H* = 1.15 m	*D* = 0.137 m; *H* = 1.37 m
IVA-01	*P* = 7.88 kW	*P* = 337 kW
T-101		1250 kW

*V* volume, *H* height, *D* diameter; *Ca* capacity, *P* power

^a^Technical specifications of equipment for a yield of 82 m^3^ of biogas ton OFMSW^−1^

It is difficult to determine a fixed yield of biogas production because the composition of OFMSW may have variations in different seasons of the year or holidays according to the experience of 3PBg. Furthermore, this part of the study considers that ADP can be used for different wholesale markets. Therefore, different possible yields that could be achieved in anaerobic digestion (70–115 m^3^ biogas ton OFMSW^−1^) are considered. It is important to mention that for this process proposal, a turbine was implemented that takes advantage of the steam generated in the drying of sludge, generating additional energy to the process, independent of the energy generated by biogas. This equipment is a direct flow steam turbine coupled to an electric generator. The equipment hosts a power generation procedure in a direct flow steam turbine generator with the main objective of expanding high-pressure steam to low-pressure steam and converting the delivered shaft power into electrical energy. In this way, electrical energy generated is used for the operation of the biogas plant and the rest is injected into the grid, together with the electrical energy coming from the Engine-Generator that treats the gas produced (IVA-01 unit). Besides, the turbine can transfer available heat to other equipment. The values of the energy yields for ADP are shown in Table 5. The results consider different yields, especially the average obtained in 3PBg of 82 m^3^ of biogas ton OFMSW^−1^.

**Table 5:** Main yield of the AD power plant

Main performance					
m^3^ Biogas ton OFMSW^−1^	Methane production (Nm^3^ d^−1^)	Biogas production (Nm^3^ d^−1^)	Power consumed (kWh d^−1^)	Power generated (MWh d^−1^)	Energy available for transfer (MW)
70	1788	3501	3444	34.4	11.8
75	1907	3748	3444	34.7	11.7
82	2080	3768	3444	35.1	11.6
90	2275	4142	3444	35.6	11.4
95	2395	4372	3444	35.9	11.4
100	2616	5016	3444	36.2	11.3
105	2638	5269	3444	36.4	11.2
110	2759	5523	3444	36.7	11.1
115	2880	5774	3444	37.0	11.0

The production of biogas, methane and electrical energy generated, increase along with the yield of biogas per ton of OFMSW. The electrical energy consumed is similar in all cases and the energy available for transfer decreases as the yield increases, since the energy comes from the turbine connected to the sludge dryer. By increasing methane production, there is less digestate, and less steam is generated to feed the turbine. The extracted heat is used as a donor for operations that require heating by means of hot water that serves as a heat transfer agent.

In contrast to 3PBg, the energy generated from ADP is higher than the energy consumed. This is due to the use of the turbine and the use of an improved Engine-Generator compared to the 3PBg. In this way, for a yield of 82 m^3^ of biogas ton OFMSW^−1^, 35.1 MWh d^−1^ is generated, in which 8.2 and 26.9 MWh d^−1^ are generated by the Engine-Generator and the turbine, respectively.

#### Economic evaluation

The process study assumed a project life of 20 years, a construction period of 30 months, a start-up phase of 4 months, and an inflation rate of 4%. The year of analysis is 2022, taking into account a 10-year depreciation period. The debt in this case is disregarded due to the uncertainty about the conditions and the amount of credit the potential investor could obtain. The net present value (NPV) is used as an economic criterion and implies the difference between the present value of cash inflows and the present value of cash outflows over a period. To determine NPV, the methodology suggested by Al-Wahaibi was used [22]. The NPV acceptance criteria in investment projects are based on the following (1) NPV > 0 accepted; (2) NPV < 0 rejected. It is important to mention that for types of projects using OFMSW as a substrate, in some regions, it is difficult to obtain a NPV > 0 [22, 56, 57]. It is usually not feasible without subsidies, because it has a negative NPV.

For this study, the values mentioned in Table 6 were used. For some of these values, it is difficult to establish a specific fixed value since there may be variations.

**Table 6:** Economic data of the process simulation

Concept	Value	Unit
*Raw material*		
Polymer	397	US $ ton^−1^
Iron (III) oxide	4130	US $ ton^−1^
NaHCO_3_	68	US $ ton^−1^
Std power^b^	0.071	US $ kWh^−1^
*Revenues*		
Tipping fee	25	US $ ton^−1^
Digestate^a^	45	US $ ton^−1^
Std power^b^	0.071	US $Wh^−1^

^a^[58]

^b^[59]

**Fig. 6:**
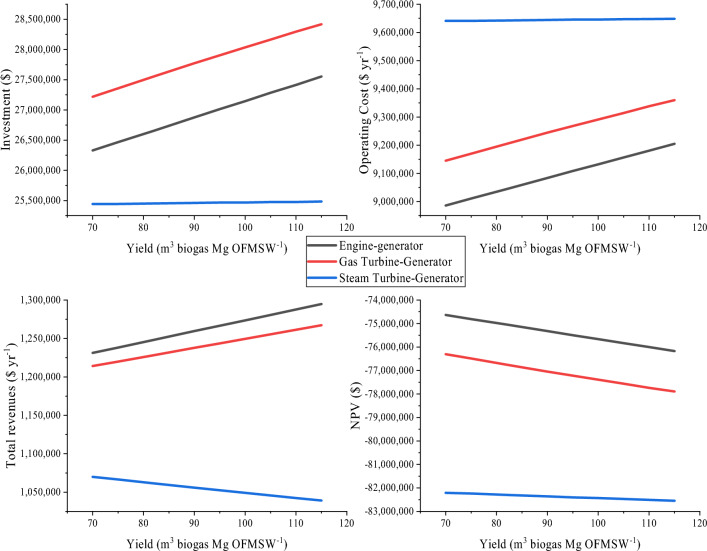
Economic summary of ADP at different yields

Figure 6 shows the effect of return on investment, operating costs, total revenues and NPV. Although our hypothesis suggested a negative NPV, it was anticipated that the increase in biogas production yield would improve the viability of the ADP project. However, the high investment values and operating costs affect the viability of ADP despite the increase in total revenues. It was expected that as heat was integrated into equipment, a high amount of energy would be generated and NPV would approach zero.

Analyzing the results of the operation, by obtaining a greater amount of biogas, a greater capacity of some equipment is required, especially those used to generate electricity. Therefore, a Gas Turbine-Generator was considered for ADP, representing a simple cycle combustion gas turbine. This is composed of an air compressor, a combustion chamber, and a gas expansion turbine, coupled to an electric generator. The turbine drives both the compressor and the generator. Finally, a steam generator was studied from the combustion of biogas in the presence of air and the sensible heat of the air and fuel inlet currents. Connected to the steam generator, a Straight-Flow Steam Turbine-Generator is used. The energy efficiency of each of the generating equipment (kW-h L_biomethane_^−1^) is 0.86, 0.84, and 0.68% for Engine-generator, Gas Turbine-Generator and Steam Turbine-Generator, respectively. The results are shown in Fig. 6.

The use of a Motor-Generator stood out for its notable economic profitability. However, when seeking to improve yield and increase gas production, it is necessary to increase the capacity of the equipment responsible for generating electrical energy. This increase in capacity leads to an increase in revenue, but also requires significantly greater investment. Since the investment required exceeds, the income generated, it is not possible to achieve a positive Net Present Value (NPV) on its own. Therefore, to achieve the economic viability of this project, it is essential to have a subsidy that supports the operation of the plant. This will allow it to be kept in operation and take advantage of its importance both in waste treatment and in the generation of electrical energy, thus benefiting wholesale markets*.* Table 7 shows an approximation of the amount of subsidy that is required for each of the yields of the study considering the use of Engine-Generator to treat biogas.

**Table 7:** Sufficient subsidy amount to achieve NPV = 0

Yield (m^3^ of biogas ton OFMSW^−1^)	Total subsidy ($)	Subsidy on the total investment (%)
70	5,703,632	21.7
75	5,717,570	21.6
82	5,731,635	21.5
90	5,742,315	21.4
95	5,751,806	21.3
100	5,764,547	21.2
105	5,773,773	21.2
110	5,786,809	21.1
115	5,795,858	21.0

The presented values in Table 7, represent the thresholds required to attain an NPV = 0, signifying a break-even point in the process. In processes geared toward achieving social benefits, such as this one, a neutral NPV can suffice. This approach aids in problem-solving while also yielding some advantages. In this scenario, the process contributes significantly to waste treatment and concurrently generates substantial electrical energy.

## Conclusions

The relevant part of this investigation was to use a single-stage pilot plant, which treats high amounts of OFMSW for 524 days. Overall, the values obtained in the pilot plant were very similar to those of other studies. In general, the yields during operation remained within the limits of acceptability, with the VS, TS and COD feed showing a normal distribution. Based on the experience of the pilot plant, we deduce the importance of considering resources to cover investment costs. Furthermore, there is a need to promote a culture of waste separation among the citizens, with the aim of enhancing the efficiency and yield of the anaerobic digestion process. Likewise, it is suggested to explore processing alternatives for obtaining value-added by-products, as well as integrating energy into the process and connecting the generated energy and produced biogas to electrical and natural gas networks.

On the other hand, a theoretical scaling study was carried out to analyze the economic profitability of a large-scale plant that treats OFMSW from wholesale markets. It is recommended to use a Motor-Generator to generate electricity, but a subsidy of around 21% of the total investment is required to achieve NPV = 0. Although it is difficult to find an economic feasibility for this process using OFMSW, the plant can make a great contribution as it can treat large quantities of organic waste and at the same time contribute to the energy demand of both the ADP process and the wholesale market. This work has provided experimental and theoretical information to support the viability of anaerobic digestion plants using waste from wholesale markets in Mexico, which could also be beneficial for other developing countries**.**

### Supplementary Information

Below is the link to the electronic supplementary material.

Supplementary file1 (DOC 78 KB)

## Data Availability

Data and materials are available upon request.
