# Supplementary material for: Wet anaerobic digestion of organic fraction of municipal solid waste: experience with long-term pilot plant operation and industrial scale-up
Source: Bioprocess Biosyst Eng. 2024 Jan 3;47(2):235–47. doi: 10.1007/s00449-023-02958-2 (PMC10867089; doi:10.1007/s00449-023-02958-2)
Supplement: Supplementary file 1 — Supplementary file1 (DOC 78 KB) [file 449_2023_2958_MOESM1_ESM.doc]

Supplementary Material

Biochemical potential of methane

The determination of the biochemical potential of methane (BMP) involved carrying out laboratory tests, such as anaerobic digestibility tests, where the amount of methane produced during a given period was measured. These tests allowed estimating the theoretical amount of methane that can be obtained from a substrate under optimal anaerobic fermentation conditions. The results generated by this test are the basis for defining the treatment of a given substrate by anaerobic means. The tests were set up in an Automatic Methane Potential Test System (AMPTS) equipment using a 2:1 mass ratio of VS and substrate, in 500 mL glass digesters hermetically sealed with nitrogen gas to guarantee anaerobic conditions. A micronutrient solution and a basal medium were added to ensure alkalinity control. Fig. 7 shows the results obtained in the experiments carried out with samples of organic waste.


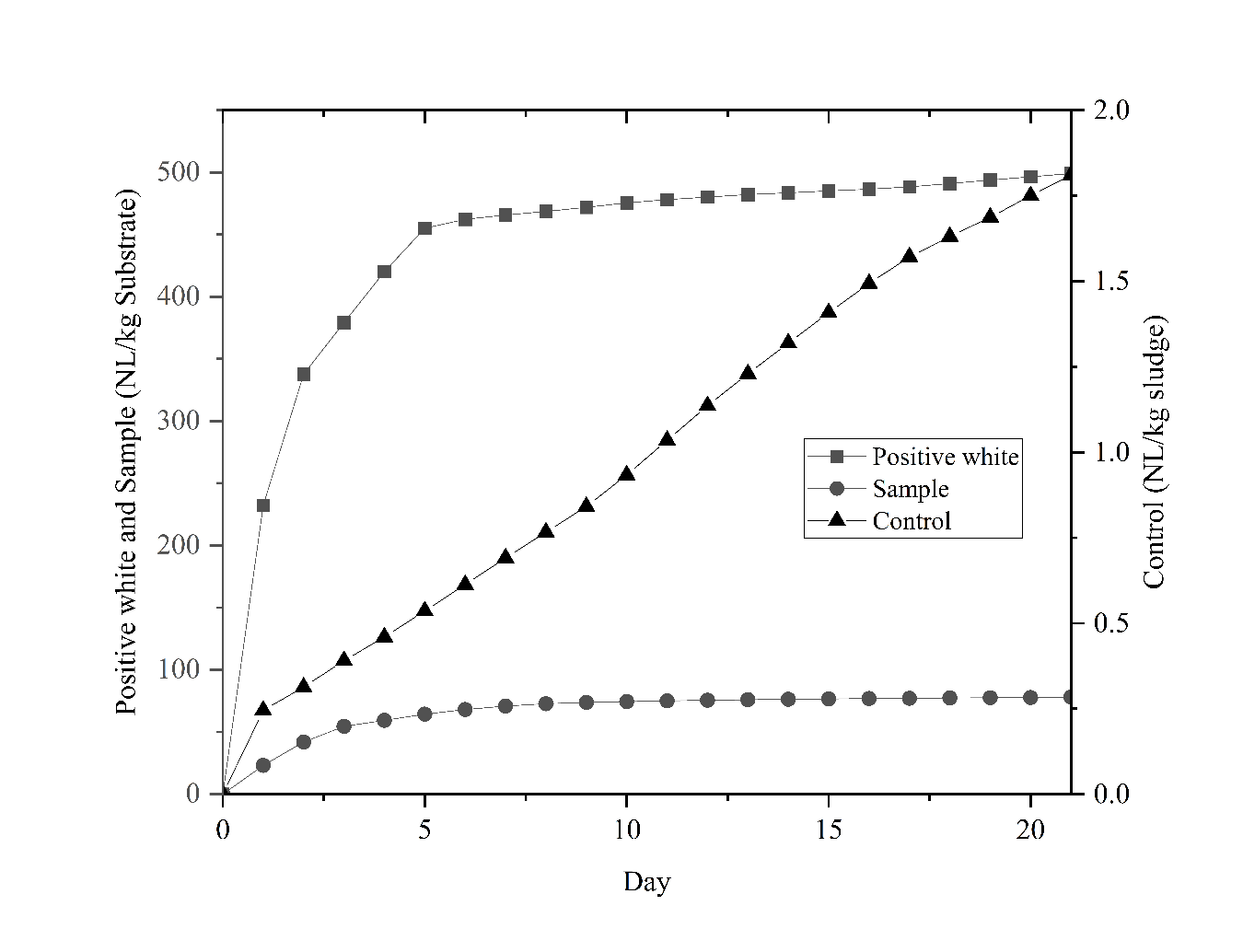


**Fig. 7**. Specific biogas production by BMP test

The curve that corresponds to the positive white, was obtained with a mixture formed with digestate from 3PBg as inoculum and completely biodegradable dextrose, managing to produce 499 NL/kg dextrose. The curve that corresponds to the control, was formed with sludge as inoculum and in turn as a substrate, obtaining a maximum production of 1.8 LN/kg sludge, where it is observed that the biogas production of the sludge was 99.6% lower compared to dextrose as substrate. Finally, the curve corresponding to the OFMSW sample had a production of 78 NL/kg OFMSW, reaching 16% of that produced with dextrose. It is important to highlight that the biochemical potential of methane can vary according to the type of substrate used, its chemical composition and the fermentation conditions. Some substrates, such as organic food waste or sewage sludge, often have a high biochemical methane potential and are commonly used in biogas production as renewable energy sources. Table 7 shows some of the characteristics obtained in this test.

**Table 7**

Characteristics of OFMSW and sludge

| Characteristic | OFMSW | Sludge |
| --- | --- | --- |
| COD (g O_2_ L^-1^) | 290 ± 7.0 | 150 ± 2.0 |
| TS (%) | 12.6 ± 0.21 | 2.3 ± 0.02 |
| VS (%) | 11.9 ± 0.22 | 1.4 ± 0.04 |
| N-NH_4_ (g N L^-1^) | 0.74 ± 0.26 | 1.4 ± 0.07 |
| P total (g P L^-1^) | 25 ± 0.74 | 2.8 ± 0.06 |
| Alkalinity (g CaCO_3_ L^-1^) | 4.5 ± 0.21 | 7.8 ± 0.33 |
| VFA (g CH_3_COOH L^-1^) | 7.6 ± 0.68 | 0.46 ± 0.12 |
| SV ST^-1^ (%) | 94 ± 0.21 | 61 ± 0.92 |
| Alpha α | 0.2 ± 0.01 | 0.91 ± 0.03 |
